# Preparation of Pt/γ-Bi_2_MoO_6_ Photocatalysts and Their Performance in α-Alkylation Reaction under Visible Light Irradiation

**DOI:** 10.3390/nano10040646

**Published:** 2020-03-30

**Authors:** Haiying Li, Xiujuan Yu, Xueli Hao, Zhiying Zhang, Yan Wang, Jingyi Li

**Affiliations:** 1 College of Chemistry and Chemical Engineering, Inner Mongolia University, Hohhot 010021, China; lihaiying0528gsj@163.com (H.L.); yuxiujuan810@163.com (X.Y.); hxl18747721020@163.com (X.H.); zhangzhiying2019@163.com (Z.Z.); wynmgnhy@163.com (Y.W.); 2 Hebei Key Laboratory of Neuropharmacology, Hebei North University, Zhangjiakou 075000, China

**Keywords:** Pt/γ-Bi_2_MoO_6_, visible light irradiation, alpha alkylation reaction

## Abstract

Bi(NO_3_)_3_·5H_2_O and (NH_4_)_6_Mo_7_O_24_·4H_2_O were used as precursors to synthesize flaky γ-Bi_2_MoO_6_ samples by a hydrothermal method, and Pt/γ-Bi_2_MoO_6_ samples with different mass fractions were prepared by an NaBH_4_ reduction method. Alpha alkylation of benzyl alcohol and acetophenone with photocatalysts under visible light irradiation was performed, and the activity of 4 wt % Pt/γ-Bi_2_MoO_6_ (γ-Bi_2_MoO_6_ was prepared by a nitric acid method, pH = 9, and reaction temperature 180 °C) was the best. The photocatalytic reaction conditions were optimized by changing various kinds of variables, such as the type of catalyst, solvent, and base, and the amount of base, catalyst, and reactant. The optimal conditions for the organic reaction were 75 mg 4 wt % Pt/γ-Bi_2_MoO_6_, 6 mL n-heptane, 1.2 mmol NaOH, 1 mmol acetophenone, and 3 mmol benzyl alcohol. Under the optimal reaction conditions, the effects of different light wavelengths and light intensities on the reaction were measured, and the cycling ability of the photocatalyst was tested. After five cycles, the photochemical properties of the catalyst were relatively stable. Finally, the active substances were identified (such as electrons (e^−^), holes (h^+^), hydroxyl radicals (•OH), and superoxide radicals (•O_2_^−^).

## 1. Introduction

Dehydration of an alpha alkylation reaction is one of the most important methods to build C=C bonds. It is significant in organic synthesis and industrial production. Traditional alkylation [1] is the reaction of organic compounds with halo-hydrocarbons and their derivatives. Although this method has a high yield, the required temperature is high, and there are many byproducts. Thus, traditional alkylation is not in line with the concept of environmental protection and energy conservation. In our experiment, benzyl alcohol was used as an alkylating agent, and an alkyl group was introduced onto acetophenone to construct C=C bonds and to synthesize 1,3-diphenyl-2-propen-1-one. Due to the low toxicity of alcohol compounds [2], the advantages of the above method producing clean products and a high atomic efficiency are significant.

Bismuth catalysts have been widely studied due to their unique photocatalytic activity, narrow band gap, and thermal stability [3,4]. Bi_2_MoO_6_ [5,6,7,8,9,10] is considered an ideal photocatalyst in bismuth catalysts. Bi_2_MoO_6_ photocatalysts have three different crystal forms: α-Bi_2_MoO_6_, β-Bi_2_MoO_6_, and γ-Bi_2_MoO_6_. Each crystal form has its own structure and property, with γ-Bi_2_MoO_6_ demonstrating a strong photoelectric effect and a wide range of visible light absorption [11]. The γ-Bi_2_MoO_6_ catalyst has a unique [MoO_6_] octahedral sandwich structure composed of a [Bi_2_O_2_]^2+^ layer and a [MoO_4_]^2−^ layer. The [MoO_4_]^2−^ layer is located at the eight corners of the octahedron, and the [Bi_2_O_2_]^2+^ layer is embedded in the middle of the octahedron [12,13]. To date, a variety of synthetic methods of γ-Bi_2_MoO_6_ have been discovered, such as the hydrothermal method [14,15], coprecipitation method [16,17], solvothermal method [18,19], and solid-phase reaction method [20]. However, the crystal form, morphology, structure, and particle size of γ-Bi_2_MoO_6_ are greatly affected by temperature; thus, the above properties are difficult to control. Therefore, a hydrothermal synthesis with a new liquid-phase synthesis method is favored by more people.

Wu [21] prepared a Bi_2_Mo_3_O_12_/Bi_2_MoO_6_ heterojunction structure by a hydrothermal method under visible light irradiation to photocatalyze water and generate oxygen. Li [12] used a hydrothermal method to synthesize low-cost indium-doped Bi_2_MoO_6_ for electrochemical performance testing. The results show that the photocatalytic reduction performance of Cr(VI) is greatly improved. Although Bi_2_MoO_6_ has good application prospects under visible light irradiation, it also has many shortcomings, such as low photogenerated electron mobility and high photoelectron and hole recombination ability. As a result, the application of the photocatalyst in the visible light range is restricted.

Therefore, it is particularly significant to prepare a modified composite photocatalyst material to improve reaction efficiency. A hydrothermal method to synthesize flaky γ-Bi_2_MoO_6_ and to load precious metals on its surface is applied to improve photocatalytic activities. Common supported metals are Pt [22,23], Pd [24,25], Au [26], Ag [27], etc. Among them, the loading of Pt particles is the most common. The loading of Pt effectively increases the separation of the photogenerated electrons and holes and improves the photocatalytic activity. The photocatalytic activity of Pt-modified Bi_2_MoO_6_ (a conversion of 80.7% under visible light irradiation for 24 h, using 6 mL of n-heptane, 1.2 mmol of NaOH, and 1:3 mmol of acetophenone and benzyl alcohol) is much higher than that of unmodified Bi_2_MoO_6_ (a conversion of 37.6%). The Fermi level of Pt nanoparticles is more negative than that of Bi_2_MoO_6_. When they are in contact, photogenerated electrons are transferred from Bi_2_MoO_6_ with a high Fermi level to the Pt surface with a low Fermi level, which effectively suppresses the recombination of electrons and holes. Furthermore, Pt is deposited on the catalyst surface in the form of atomic clusters. When the loading of Pt is too high, electrons and holes will be recombined rapidly [28], which is not conducive to the α-alkylation reaction.

## 2. Materials and Methods

### 2.1. Preparation of the γ-Bi_2_MoO_6_

#### 2.1.1. Preparation of γ-Bi_2_MoO_6_ Using the Nitric Acid Method

A simple hydrothermal method was used to prepare pure γ-Bi_2_MoO_6_. All chemicals were of analytical grade and were used without further purification. At room temperature, 2 mmol of Bi(NO_3_)_3_·5H_2_O was dissolved in 25 mL [29] of 2 M nitric acid solution and then stirred on a magnetic stirrer until it was completely dissolved to form solution A. Moreover, 0.14 mmol of (NH_4_)_6_MO_7_O_24_·4H_2_O was dissolved in 30 mL of deionized water and stirred until it was completely dissolved to form solution B. Next, solution B was slowly dropped into solution A, and then the solution was mixed and stirred for 20 min. Next, the pH was adjusted to 8, 9, 10, and 11 with ammonia water. The pH-adjusted mixture was transferred to a 100 mL autoclave and heated at 180 °C (150 °C or 130 °C) for 12 h. Then, the mixture was cooled naturally to room temperature and finally used. The samples were washed with deionized water and absolute ethanol several times and dried at 80 °C for 12 h. Finally, γ-Bi_2_MoO_6_ was obtained after sufficient grinding.

#### 2.1.2. Preparation of γ-Bi_2_MoO_6_ Using the Ethylene Glycol Method

At room temperature, 2 mmol of Bi(NO_3_)_3_·5H_2_O was dissolved in 25 mL of 2 M ethylene glycol solution and stirred on a magnetic stirrer until it was completely dissolved to form solution A. Moreover, 0.14 mmol of (NH_4_)_6_MO_7_O_24_·4H_2_O was dissolved in 30 mL of deionized water and continuously stirred until it was completely dissolved to form solution B. Next, solution B was slowly dropped into solution A, and then the solution was mixed and stirred for 20 min. Next, the pH was adjusted to 9 with concentrated ammonia water. Then, the pH-adjusted mixture was transferred to a 100 mL autoclave and heated at 180 °C for 12 h. Afterward, the sample was cooled naturally to room temperature. Deionized water and anhydrous ethanol were used to wash the sample several times, and then it was dried at 80 °C for 12 h. Finally, γ-Bi_2_MoO_6_ could be obtained after sufficient grinding.

#### 2.1.3. Preparation of γ-Bi_2_MoO_6_ Using the Glacial Acetic Acid Method

At room temperature, 2 mmol of Bi(NO_3_)_3_·5H_2_O was dissolved in 25 mL of 2 M glacial acetic acid solution and stirred on a magnetic stirrer until it was completely dissolved to form solution A. Additionally, 0.14 mmol of (NH_4_)_6_MO_7_O_24_·4H_2_O was dissolved in 30 mL of deionized water and continuously stirred until it was completely dissolved to form solution B. Next, solution B was slowly dropped into solution A, and then the solution was mixed and stirred for 20 min. The pH was adjusted to 9 with ammonia water. Then, the pH-adjusted mixture was transferred to a 100 mL autoclave and heated at 180 °C for 12 h. Next, the sample was cooled naturally to room temperature. Deionized water and anhydrous ethanol were used to wash the sample several times, and then it was dried for 12 h at 80 °C. Finally, γ-Bi_2_MoO_6_ could be obtained after sufficient grinding.

### 2.2. Preparation of Pt/γ-Bi_2_MoO_6_ by the NaBH_4_ Reduction Method

An analytical balance was used to weigh 2.5 g of the carrier, and it was dissolved in 200 mL of deionized water and sonicated for 30 min. Then, 5 wt % PEG2000 was added, and the solution was stirred for 10 min and sonicated for 10 min. A certain amount of H_2_PtCl_6_ (0.00765 M) was added dropwise for 20 min to the mixed solution in a separating funnel. A pipette was used to add 20 mL of the lysine solution, which was added dropwise for 10 min, and stirred for 10 min. Then, 0.135 g of NaBH_4_ was dissolved in 10 mL of water and added dropwise to the above solution before being stirred for 10 min. Next 10 mL of HCl (0.3 M) was pipetted into the above solution dropwise for 10 min, stirred for 1 h and aged for 24 h, The resulting catalysts were washed three times with deionized water and ethanol, and dried at 60 °C for 12 h to obtain 1 wt %, 2 wt %, 3 wt %, 4 wt %, and 5 wt % Pt/γ-Bi_2_MoO_6_.

### 2.3. Characterization Method

The crystal phase and structure of the catalyst were measured using a D/MAX-2500 powder X-ray diffractometer (XRD, Rigaku Industrial Corporation, Osaka, Japan). The scanning range of the sample was 5–80°, and the scanning speed was 1°/min with a scanning step of 0.05°. The voltage in the tube was 40 kV. The current in the tube was 100 mA, and a copper target was used as the metal target (*λ* = 1.5405 Å). The metal and chemical valence values on the surface of the catalyst were measured by X-ray photoelectron spectroscopy (XPS, Thermo Fisher Scientific Company, Waltham, MA, USA) with an ESCLAB-250Xi instrument, and the beam spot diameter of the monochromatic light X-ray source was 200–900 μm. The morphology of the catalyst was measured by scanning electron microscopy (SEM, Hitachi Limited Company, Tokyo, Japan) with an S-4800 microscope, and the high pressure was 10 kV. The internal structure and interplanar spacing of the catalyst were measured using field emission transmission electron microscopy (TEM, FEI Tecnai F20, FEI Company, Hillsborough, OR, USA), and its acceleration voltage was 200 kV. The light absorption range of the catalyst was measured by UV-vis diffuse reflectance spectroscopy (UV-vis DRS, Hitachi Limited Company, Tokyo, Japan) using a U-390 solid ultraviolet-visible absorption spectrometer, and its scanning wavelength was 200–800 nm. The specific surface size and pore size distribution of the catalyst were measured by N_2_ adsorption desorption isotherm curves (Brunauer-Emmett-Teller (BET), Quantachrome Instruments, Corporate Headquarters, Boynton Beach, FL, USA). N_2_ was used as the analysis gas, and the bath temperature was 77.35 K. The photoluminescence (PL, Edinburgh Instruments, Edinburgh, Scot., British) spectrum of the catalyst was measured by a FLS920 fluorescence spectrometer. The light source was a pulsed xenon lamp (450 W), the excitation wavelength was 275 nm, and the wavelength range was 400–550 nm. The conversion and selectivity of the reaction were measured using a gas chromatograph (GC-2014C, Shimadzu Company, Kyoto, Japan) with comparisons to analytical grade standard samples. A gas chromatograph-mass spectrometer (GC–MS, Finnigan Company, Silicon valley, CA, USA) was used to identify the reactants and products. The results of GC-MS were shown in Supplementary Information.

## 3. Results and Discussion

### 3.1. X-ray Diffraction (XRD) Analysis

**Figure 1:**
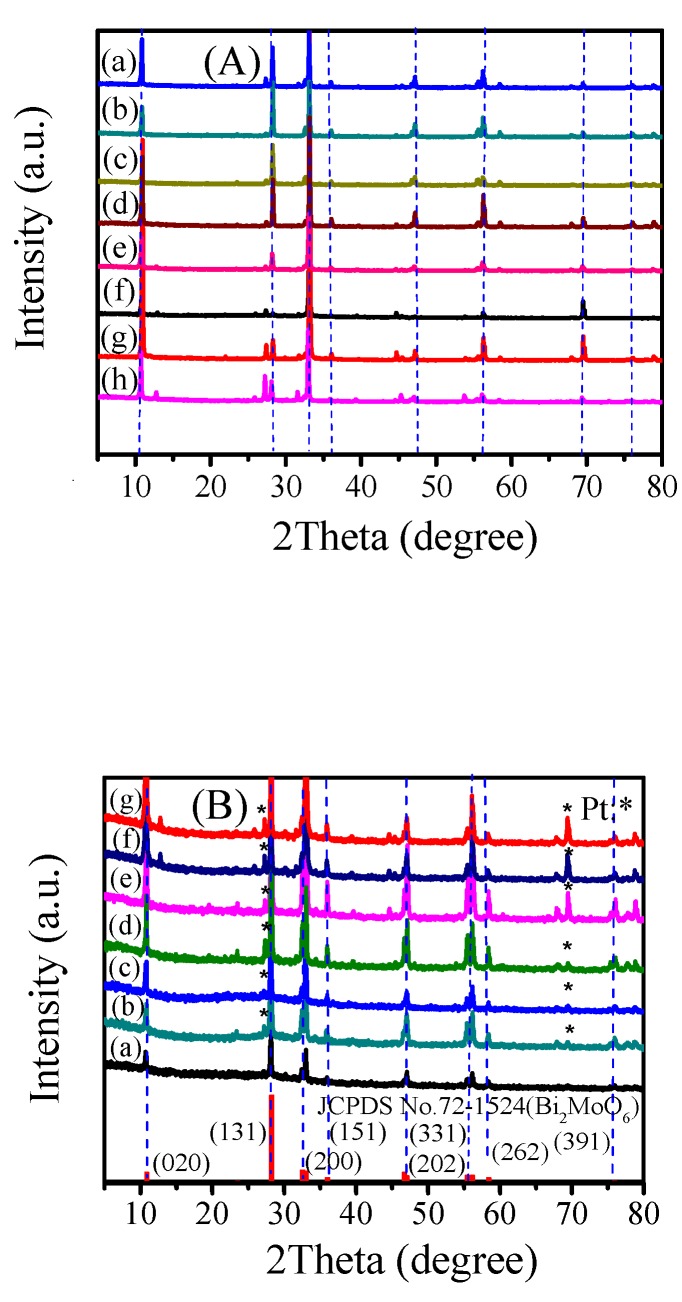
(**A**) XRD patterns of different catalysts: (**a**–**c**) γ-Bi_2_MoO_6_ (ethylene glycol method, nitric acid method, and glacial acetic method, pH = 9, 180 °C), (**d**–**e**) γ-Bi_2_MoO_6_ (nitric acid method, pH = 9, 150 °C, and 130 °C), (**f**–**h**) γ-Bi_2_MoO_6_ (nitric acid method, pH = 8, 10, and 11, 180 °C). (**B**) XRD patterns of different catalysts: (**a**) γ-Bi_2_MoO_6_ (nitric acid method, pH = 9, 180 °C), (**b**–**f**) 1 wt %, 2 wt %, 3 wt %, 4 wt %, and 5 wt % Pt/γ-Bi_2_Mo_6_ (nitric acid method, pH = 9, 180 °C), and (**g**) recycled 4 wt % Pt/γ-Bi_2_MoO_6_ (nitric acid method, pH = 9, 180 °C).

According to the standard card (JCPDS, No.72-1524, γ-Bi_2_MoO_6_), γ-Bi_2_MoO_6_ was successfully prepared (Figure 1). The positions of the diffraction peaks of γ-Bi_2_MoO_6_ obtained under different conditions were basically the same, but the peak intensity changed. The 2*θ* values at 10.8°, 28.2°, 32.5°, 36.0°, 46.7°, 55.5°, 58.4°, and 75.9° corresponded to the (020), (131), (200), (151), (202), (331), (262), and (391) crystals of γ-Bi_2_MoO_6_, respectively. When γ-Bi_2_MoO_6_ was supported with different mass fractions of Pt, new diffraction peaks appeared, indicating that the Pt was successfully loaded onto γ-Bi_2_MoO_6_ (Figure 1B). The intensity of the diffraction peaks of Pt increased with an increasing mass fraction of Pt. The peak strength did not change significantly after five cycles (Figure 1Bg). This result verified that 4 wt % Pt/γ-Bi_2_MoO_6_ was stable, but it might also be because the crystals of γ-Bi_2_MoO_6_ were not damaged.

### 3.2. X-ray Photoelectron Spectroscopy (XPS) Analysis

**Figure 2:**
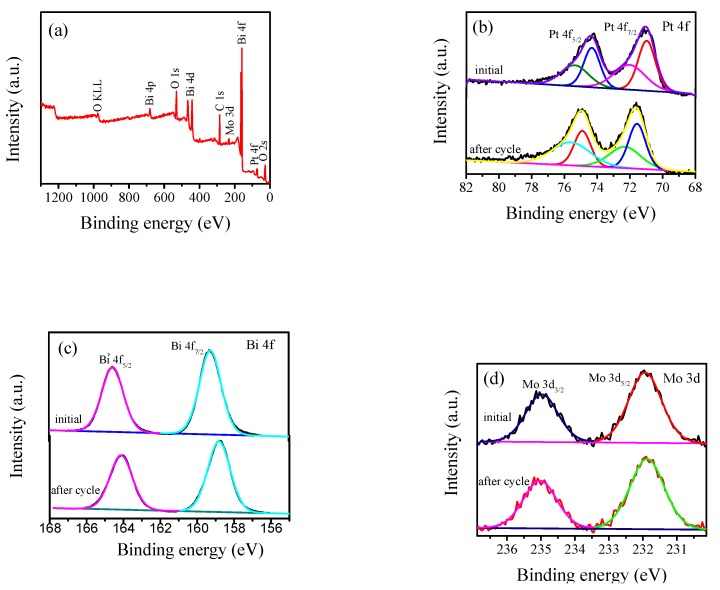

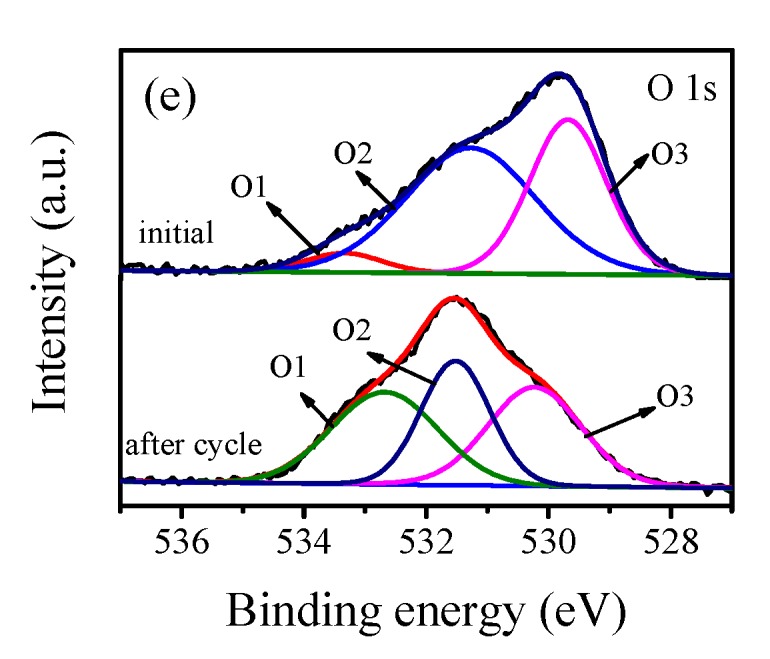
XPS spectra of 4 wt % Pt/Bi_2_MoO_6_ (nitric acid method, pH = 9, 180 °C) before and after cycling: (**a**) full spectrum, (**b**) Pt 4f, (**c**) Bi 4f, (**d**) Mo 3d, and (**e**) O 1s.

The elemental chemical states of the prepared samples were analyzed by XPS. Figure 2a shows the full spectrum of the 4 wt % Pt/γ-Bi_2_MoO_6_ photocatalyst. Pt/γ-Bi_2_MoO_6_ of 4 wt % (nitric acid method, pH = 9, 180 °C) is mainly composed of Pt, Bi, Mo, and O. Figure 2b–e shows the high-resolution XPS spectra [30] of Pt, Bi, Mo, and O. Figure 2b shows the 4f spectra of Pt before and after cycling of the 4 wt % Pt/γ-Bi_2_MoO_6_ photocatalyst, and the peaks at 71.15 and 74.52 eV corresponded to the binding energies of Pt^0^ 4f_7/2_ and Pt^0^ 4f_5/2_ before cycling, respectively. The peaks at 72.27 and 76.31 eV corresponded to the binding energies of Pt^2+^ 4f_7/2_ and Pt^2+^ 4f_5/2_ before cycling, respectively. The peaks at 71.69 and 75.04 eV corresponded to the binding energies of Pt^0^ 4f_7/2_ and Pt^0^ 4f_5/2_ after cycling, respectively. The peaks at 73.68 and 77.03 eV corresponded to the peaks of Pt^2+^ 4f_7/2_ and Pt^2+^ 4f_5/2_ after cycling, respectively. The binding energy indicates that Pt had two valence states of 0 and +2 on the surface of the catalyst, and some of the Pt^0^ was oxidized to Pt^2+^. Two typical peaks of Bi^3+^ are shown in Figure 2c. The peaks at 158.82 and 164.12 eV corresponded to the binding energies of Bi^3+^ 4f_7/2_ and Bi^3+^ 4f_5/2_ before the cycle, respectively. The peaks at 159.25 eV and 164.55 eV corresponded to the binding energies of Bi^3+^ 4f_7/2_ and Bi^3+^ 4f_5/2_ after the cycle, respectively. In Figure 2d, there were two peaks of Mo^6+^ at 231.87 and 235.03 eV and they corresponded to the binding energies of Mo^6+^ 3d_5/2_ and Mo^6+^ 3d_3/2_ before cycling, respectively. The peaks at 231.96 and 235.23 eV [31] corresponded to the binding energies of Mo^6+^ 3d_5/2_ and Mo^6+^ 3d _3/2_ after cycling, respectively. The binding energies of Bi and Mo were shifted, which may be related to the changes in the chemical environment of Bi and Mo. Figure 2e shows the O 1s spectrum. The binding energies before the cycle were located at 533.34 (O1), 531.27 (O2), and 529.68 eV (O3). The binding energies after the cycle were located at 532.68 (O1), 531.52 (O2), and 530.23eV (O3). O1 represents the oxygen formed by the hydroxide adsorbed on the catalyst surface, O2 represents the [MoO_4_]^2−^ layer of γ-Bi_2_MoO_6_, and O3 represents the lattice oxygen of the [Bi_2_O_2_]^2+^ layer; furthermore, O3 of γ-Bi_2_MoO_6_ in the O 1s spectrum was significantly reduced after the fifth cycle, which indicates that the [Bi_2_O_2_]^2+^ layer lattice oxygen played a major role. Thus, the decreased conversion was closely related to the reduction of O3.

### 3.3. Scanning Electron Microscope (SEM) Analysis

**Figure 3:**
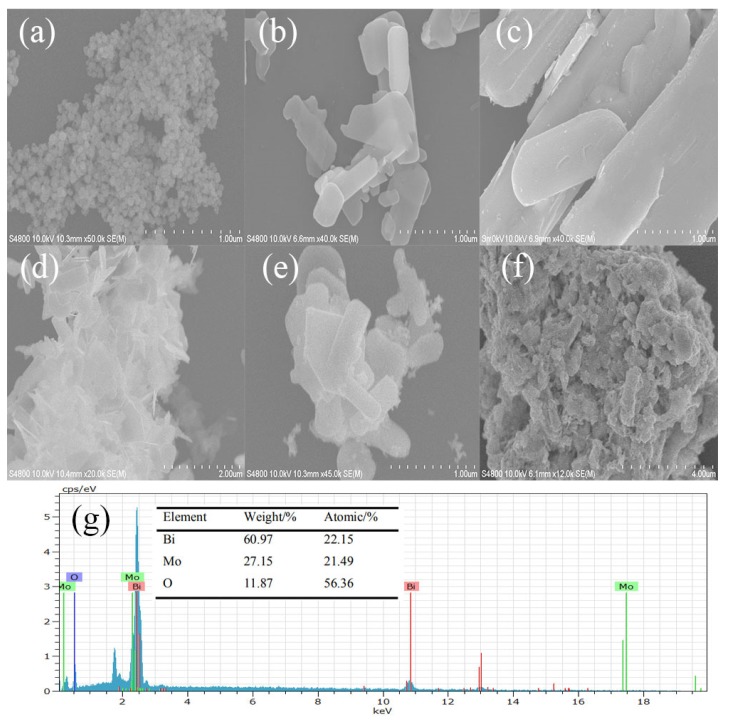
SEM images of the as-prepared samples: (**a**–**d**) γ-Bi_2_MoO_6_ (pH = 8, 9, 10, and 11, nitric acid method, 180 °C), (**e**) 4 wt % Pt/γ-Bi_2_MoO_6_ (nitric acid method, pH = 9, 180 °C), (**f**) recycled 4 wt % Pt/γ-Bi_2_MoO_6_ (nitric acid method, pH = 9, 180 °C), and (**g**) the corresponding EDX spectra of γ-Bi_2_MoO_6_ (nitric acid method, pH = 9, 180 °C).

Figure 3 is an SEM image of the as-prepared samples_._ From Figure 3g, there were three elements of Bi, Mo, and O in γ-Bi_2_MoO_6_. This was consistent with the XRD results, where γ-Bi_2_MoO_6_ was successfully prepared. SEM images of the prepared γ-Bi_2_MoO_6_ with different pH values were flakes, and the thickness of the flakes was different in Figure 3a–d. In Figure 3b, γ-Bi_2_MoO_6_ prepared by the nitric acid method and pH = 9 was the thinnest. The thinner the nanosheets are, the easier it is to generate holes and transfer electrons to the surface [32], which is beneficial when the nanosheets are in contact with reactants; thus, the conversion rate of the reaction improved. The photocatalytic performance of γ-Bi_2_MoO_6_ (pH = 9) was the best. Pt was successfully loaded on the support, as shown in Figure 3e. Figure 3f is an SEM image after five cycles. It can be seen that the stacking of nanosheets resulted in a decrease in the contact surface between the catalyst and the reactants, which reduced the reaction activity.

### 3.4. Transmission Electron Microscopy (TEM) Analysis

**Figure 4:**
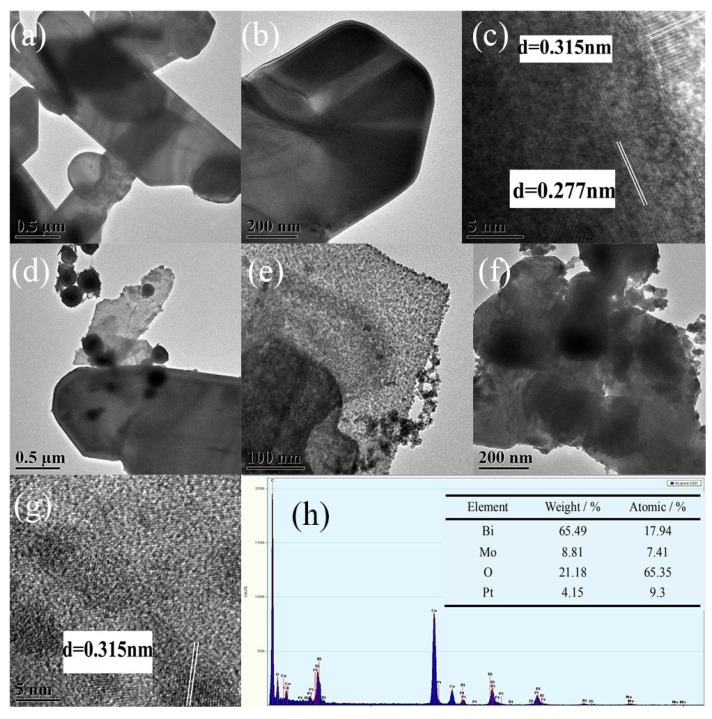
TEM images of the as-prepared samples: (**a**–**c**) γ-Bi_2_MoO_6_ (nitric acid method, pH = 9, 180 °C), (**d**–**e**) 4 wt % Pt/γ-Bi_2_MoO_6_ (nitric acid method, pH = 9, 180 °C), (**f**–**g**) recycled 4 wt % Pt/γ-Bi_2_MoO_6_ (nitric acid method, pH = 9, 180 °C), and (**h**) the corresponding EDX spectra of 4 wt % Pt/γ-Bi_2_MoO_6_ (nitric acid method, pH = 9, 180 °C).

TEM can characterize the internal structure of the catalyst. Figure 4a,b shows a flaky γ-Bi_2_MoO_6_ with the nitric acid method that was at pH = 9 and utilized a hydrothermal temperature of 180 °C. The nanoflakes were very thin. This was consistent with the SEM results. Figure 4c shows a high-resolution electron microscopy image of γ-Bi_2_MoO_6_ with particle size intervals of 0.277 nm and 0.315 nm that corresponded to the (200) and (131) crystal planes of γ-Bi_2_MoO_6_ in the orthogonal crystal system. Pt nanoparticles were successfully supported on γ-Bi_2_MoO_6_, as shown in Figure 4d,e. Therefore, the visible light region was expanded and the recombination probability of photogenerated holes and electrons was reduced. Furthermore, Pt could act as an electron trapping agent; compared with the monomer γ-Bi_2_MoO_6_, Pt/γ-Bi_2_MoO_6_ was more effective. The Pt/γ-Bi_2_MoO_6_ catalyst shows a high reactivity, and it can be seen from Figure 4h that the Pt weight loading was 4.15%, which was close to the theoretical value. The above result was consistent with the XRD and SEM results. However, when the loading of Pt was too high, Pt will agglomerate, which results in a decrease in the active sites of the catalyst and a decrease in the activity of the reaction (TEM image of 5 wt % Pt/γ-Bi_2_MoO_6_ was omitted). Figure 4f,g shows a TEM and HRTEM image after five cycles. It can be seen that the sample had only (131) crystal planes, the crystallinity of the catalyst decreased, and the number of active sites decreased, which resulted in a decrease in the catalytic activity.

### 3.5. UV-vis Diffuse Reflectance Spectroscopy (DRS) Analysis

**Figure 5:**
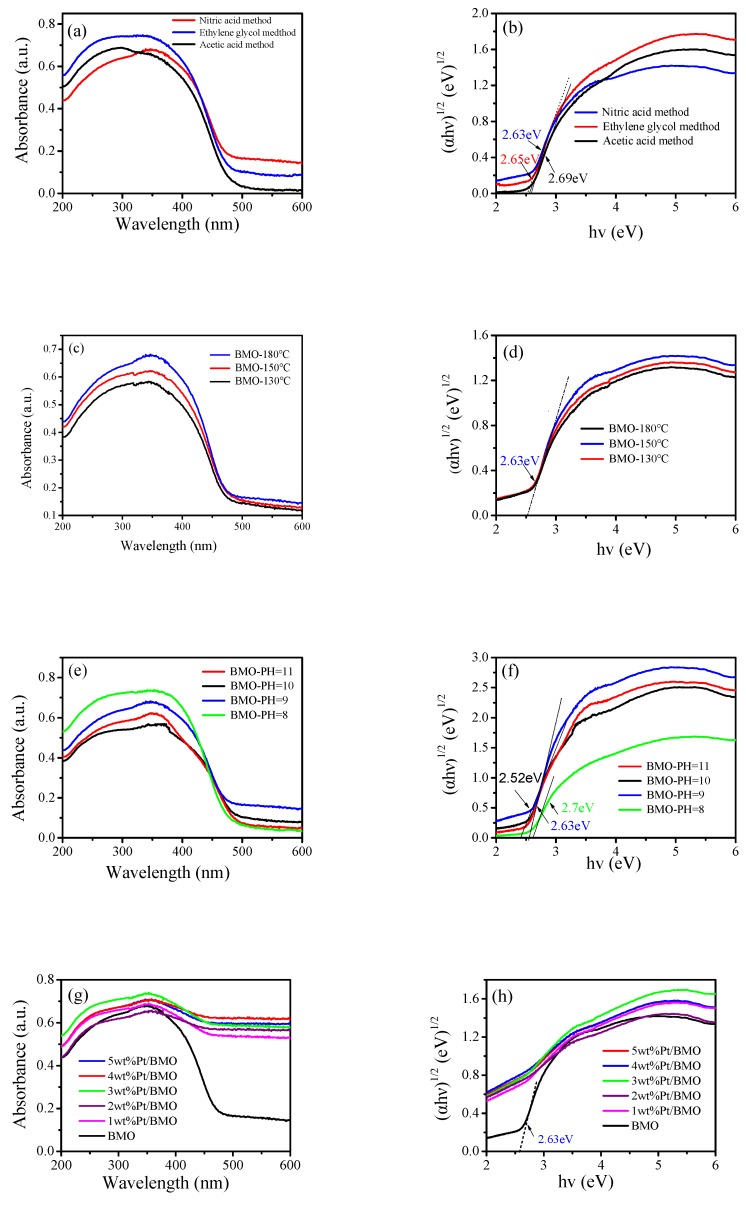
UV-vis diffuse reflectance spectroscopy (DRS) patterns of γ-Bi_2_MoO_6_ with: (**a**,**b**) different preparation methods, **(c,d**) different hydrothermal temperatures, and (**e**,**f**) different pH values and UV-vis DRS patterns of Pt/γ-Bi_2_MoO_6_ with (**g**,**h**) different mass fractions of Pt.

The UV-visible diffuse reflectance spectrum of the photocatalyst is shown in Figure 5. The absorption edge of pure bismuth molybdate prepared by different methods was approximately 480 nm, indicating that the sample had an absorption in the visible light region. The energy band gap of a semiconductor photocatalyst followed the equation αhυ = A(hυ − Eg)^n/2^, where α, h, υ, A, and E_g_ represent the diffusion absorption, Planck’s constant, the frequency, a constant, and the band gap energy, respectively. Additionally, Bi_2_MoO_6_ had an n value of 1. According to the formula E_g_ = 1240/λ_0_, the value of the forbidden band width can be estimated, where E_g_ represents the forbidden band width of the photocatalytic material (unit: eV) and λ_0_ represents the intersection of the extension line and the abscissa of the absorption wavelength of the photocatalytic semiconductor (unit: nm), which can be calculated from Figure 5a. The forbidden band width of the γ-Bi_2_MoO_6_ photocatalyst prepared by the nitric acid method was 2.63 eV. The catalytic activity of photocatalytic semiconductors mainly depends on the forbidden band width of the photocatalyst. When the forbidden band width value is small, the absorption boundary of the semiconductor moves toward visible light, which increases the utilization of visible light. The forbidden band width value of pure bismuth molybdate prepared at different temperatures was 2.63 eV (Figure 5d). It shows that the effect of temperature change on the forbidden band width was not obvious. It can be seen from the Figure 5f that when the PH value increased, the forbidden band width gradually became narrower. The results show that a change in the pH value had an effect on the forbidden band width of the catalyst. After Pt was loaded (Figure 5g,h), the absorption boundary of the catalyst moved toward the long wave region, that is to say, a redshift phenomenon occurred. The above observation indicates that Pt/γ-Bi_2_MoO_6_ had a high utilization of visible light, which enhanced its reactivity.

### 3.6. N_2_ Adsorption–Desorption Isotherm Curves (BET)

**Figure 6:**
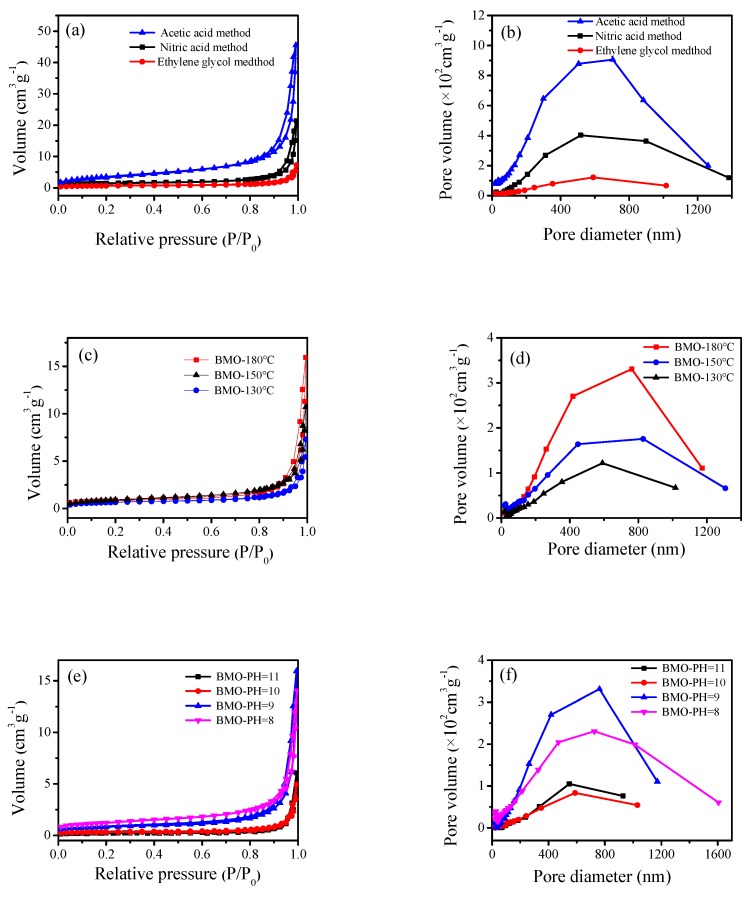

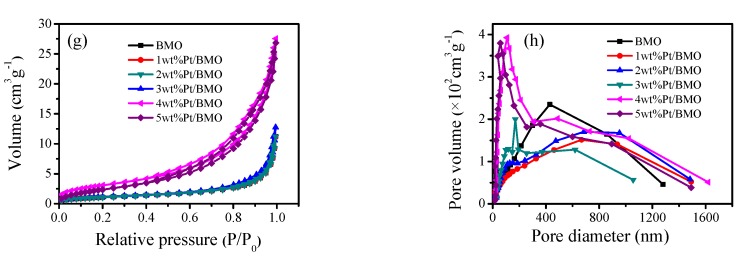
N_2_ adsorption–desorption isotherm curve (Brunauer-Emmett-Teller, BET) of γ-Bi_2_MoO_6_ with: (**a,b**) different preparation methods, (**c,d**) different hydrothermal temperatures, and (**e,f**) different pH values and N_2_ adsorption–desorption isotherm curve (BET) of Pt/γ-Bi_2_MoO_6_ with (**g,h**) different mass fraction of Pt.

The specific surface area, average pore size, and pore size distribution of the photocatalyst were determined by N_2_ adsorption–desorption isotherms. It is a type of physical adsorption. It can be seen from Figure 6a,c,e,g that the photocatalyst had type IV isotherms and type H3 hysteresis loops. H3 hysteresis loops were observed with sheet-like granular materials, which was consistent with the SEM and TEM characterization results. The maximum specific surface area (Table 1) was 13.08 m^2^·g^−1^, which might be due to the presence of a large amount of C from glacial acetic acid in the catalyst, and therefore, the specific surface area was increased. From the activity test, it was known that the correlation between the specific surface area and the activity was not large, which might be because the catalyst with a large specific surface area had a stacking phenomenon and the surface catalytic active sites were reduced. According to the IUPAC classification, pores with a pore diameter greater than 50 nm were called macropores. From Figure 6b,d,f,h, it can be seen that a macroporous structure existed in the Bi_2_MoO_6_ samples, which was conducive to enhancing the photocatalytic activity.

### 3.7. Photoluminescence (PL) Analysis

**Figure 7:**
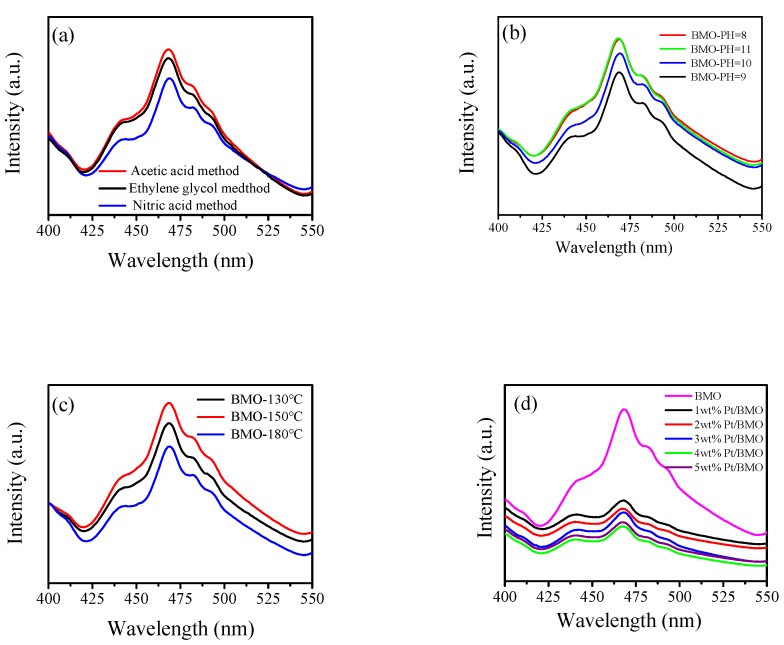
Photoluminescence spectra of γ-Bi_2_MoO_6_ with: (**a**) different preparation methods, (**b**) different pH values, and (**c**) different hydrothermal temperatures. Photoluminescence spectra of Pt/γ-Bi_2_MoO_6_ with (**d**) different mass fractions of Pt.

Figure 7 shows the photoluminescence spectrum of the as-prepared samples with an excitation wavelength of 275 nm and a wavelength range of 400–550 nm. From Figure 7a–d, it can be seen that the various preparation conditions of the semiconductor did not change the position of the emission peak. The peak signal of pure bismuth molybdate was the strongest, and the catalyst shows a weaker peak signal after being loaded with Pt, indicating that Pt as an electron trapping agent could effectively suppress the recombination probability of holes and electrons. Thus, the 4 wt % Pt/γ-Bi_2_MoO_6_ photocatalyst had the lowest recombination probability, which was consistent with the activity test of the catalyst.

### 3.8. Catalyst Activity Test

#### 3.8.1. The Alpha Alkylation Reaction of Benzyl Alcohol and Acetophenone

In this experiment, 400–800 nm filters were utilized. Benzyl alcohol and acetophenone were used as reactants, and 6 mL of solvent was added to a certain amount of base and catalyst in a 50 mL round bottom flask for the activity test. A tungsten lamp with an intensity of 2.5 × 10^−2^ W·cm^−2^ was selected as the light source. The temperature was 30 ± 3 °C and magnetic stirring was performed for 24 h. Under the above conditions, benzyl alcohol and acetophenone were added as substrates to be coupled into 1,3-diphenyl-2-propen-1-one, and the effects of different catalysts, solvents, and bases, and the amount of catalysts, bases, and reactants on the reaction was explored. At the end, the optimal reaction conditions were obtained. Finally, the content of each component was analyzed by gas chromatography, and the structure of unknown components was determined by standard calibration and GC–MS.

The equation for the alpha alkylation reaction of benzyl alcohol and acetophenone to form 1,3-diphenyl-2-propen-1-one is as follows: 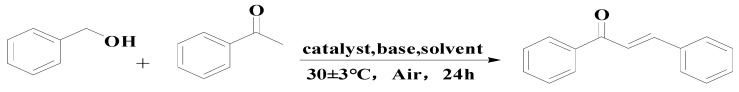


#### 3.8.2. Effect of the Different Catalysts on the Reaction

The effect of the different preparation methods, pH values, and hydrothermal temperatures of γ-Bi_2_MoO_6_ on the photocatalytic performance of the catalyst was explored, and the catalysts were loaded with different mass fractions of Pt. Thirteen different catalysts were prepared, and the α-alkylation reaction was studied. Six milliliters of n-heptane, 1 mmol of NaOH, and 50 mg of different catalysts were measured and placed into a round bottom flask. With the above materials, 1 mmol of benzyl alcohol and acetophenone acted as substrates and were coupled into 1,3-diphenyl-2-propen-1-one. The effects of the different catalysts on the α-alkylation of benzyl alcohol and acetophenone are shown in Table 2.

Among the different preparation methods, γ-Bi_2_MoO_6_ prepared by the nitric acid method was more conducive to the α-alkylation of benzyl alcohol and acetophenone (Table 2, No. 1–3). When the pH value was 9 during the preparation of γ-Bi_2_MoO_6_, the conversion rate of the reactant reached 37.6% (Table 3, No. 1, 4–6), and the conversion rate decreased when the pH value was 8 or 10–11 (Table 3, No. 4–6). This phenomenon may be caused by the accumulation of nanosheets. As the hydrothermal temperature increased, the conversion rate increased in turn, and more active sites were generated. After Pt metal was supported on pure bismuth molybdate, Pt acted as an electron capture agent, which reduced the recombination of holes and electrons. From Table 2, as the mass fraction of Pt metal increased, the reaction conversion rate increased and then decreased. Moreover, 4 wt % Pt/γ-Bi_2_MoO_6_ (Table 2, No. 12) had a conversion rate of 55.9%, indicating that the loading of Pt metal on the support could improve the conversion rate.

#### 3.8.3. Effect of the Different Amounts of Catalysts on the Reaction 

The 4 wt % Pt/γ-Bi_2_MoO_6_ (nitrate method, pH = 9, 180 °C) catalyst was selected and placed in a 50 mL round bottom flask. The other reaction conditions were the same as those described above, and the effect of different catalyst doses on the α-alkylation reaction was explored.

This experiment explored five different catalyst doses. In Table 3, the conversion rate of the light reaction was extremely low without the catalyst, and the conversion rate of the dark reaction was 0. Light and catalysts were indispensable conditions for catalyzing the α-alkylation reaction. As the amount of catalyst increased, the conversion rate first increased and then decreased. When the amount of catalyst reached 75 mg, the conversion rate was the highest (65.3%), and the selectivity did not change much. When the amount of catalyst was too large, it would lead to the excessive occupation of light-sensitive sites on the catalyst nanoparticles, which led to a decrease in the light absorption capacity of the catalyst and a reduction in the conversion rate.

#### 3.8.4. Effect of the Different Solvents on the Reaction

This experiment studied the effects of different solvents on the reaction. The conditions were the same as those above. Under the above conditions, 6 mL of different solvents were added into a 50 mL round bottom flask. The results are shown in Table 4**.**

This experiment explored the effects of 12 different solvents on the reaction. In Table 4, the conversion of the light reaction of each solvent was higher than that of the dark reaction, and the conversions of the dark reaction were very low. In the photoreaction, the conversion rate of the reaction changed with the polarity of the solvent. An increase in the polarity of the solvent reduced the conversion rate of the reaction when the polar index of the solvents was more than 4.3; however, the selectivity basically reached 100%. When the polarity of the solvent was high, the stability of the catalyst was reduced. For n-heptane, the conversion could reach 65.3%.

#### 3.8.5. Effect of the Different Bases on the Reaction

The effects of seven different bases on the reaction were investigated. Six milliliters of n-heptane and 1 mmol of base were used. The other reaction conditions were the same as those described above. The effects of different bases on the reaction are shown in Table 5.

In Table 5, the conversion rate of the light reaction was higher than that of the dark reaction. In the light reaction, the conversion rate using a strong base was significantly higher than that using a weak base, and the selectivity did not change much. The conversion rate reached 65.3% (Table 5, No. 1). However, in weak bases, the conversion rates of the reactions were very low, which might be because the reactants were sensitive to NaOH.

#### 3.8.6. Effect of the Different Amounts of the Base on the Reaction

Different amounts of 4 wt % Pt/γ-Bi_2_MoO_6_ catalysts were selected and placed in a 50 mL round bottom flask. The other reaction conditions were the same as those described above. The effects of different amounts of base on the reaction were explored, and the results are shown in Table 6.

When the amount of base was 0, the alkylation reaction could not proceed. When the amount of base was gradually increased from 0.6 to 1.2 mmol, the conversion rate gradually increased. When the amount of base reached 1.2 mmol, the conversion rate was up to 71.7%, and the selectivity was close to 100%. However, when the amount of base increased to 1.4 mmol, the conversion rate decreased because the amount of base was too high. A shielding effect occurred during the reaction, which decreased the conversion rate of the reaction.

#### 3.8.7. Effect of the Different Amounts of Reactants on the Reaction

The reaction conditions are the same as those above. The data are shown in Table 7.

The effects of six different amounts of reactants on the alkylation reaction were explored. Under light conditions, the conversion of 1 mmol of acetophenone and 3 mmol of benzyl alcohol was the highest. The conversion rate increased with the increase of the amount of benzyl alcohol, when the amount of benzyl alcohol increased to 4 mmol, the conversion rate of the reaction started to decrease, so the optimal amount of benzyl alcohol in this reaction was 3 mmol, and the selectivity approached 100%. The above observation indicates that with the increase in the amount of benzyl alcohol, more benzyl alcohol could be oxidized to benzaldehyde thereby increasing the conversion rate of the reaction. When the amount of acetophenone was increased, the conversion rate of the reaction decreased. This is because the increase in acetophenone would occupy the active site. In summary, when the molar ratio of benzyl alcohol to acetophenone was 3:1, the conversion rate of the reaction reached up to 80.7%

#### 3.8.8. Effect of the Different Alcohol and Acetophenone Derivatives on the Reaction

To extend the universality of the alkylation reaction of benzyl alcohol and acetophenone, the reaction of four different alcohol derivatives with acetophenone and benzyl alcohol and four different ketone derivatives were explored. The optimal conditions were determined after a series of experiments. In an air atmosphere, 75 mg of 4 wt % Pt/γ-Bi_2_MoO_6_, 6 mL of n-heptane, 1.2 mmol of NaOH, 3 mmol of alcohol, and 1 mmol of ketone as reactants were added to a 50 mL round bottom flask and stirred for 24 h. During the reaction process, a 400–800 nm filter was added between the light source and the round bottom flask. The results are shown in Table 8.

The conversion rate was related to the fatty alcohol connected to the benzene ring. When a saturated fatty alcohol was connected to the benzene ring, the conversion rate was low, and the conversion rate of the linear alcohol increased with the increase in the number of carbon atoms. The conversion rate of the alkylation reaction of the acetophenone derivative with an electron-withdrawing group (–Cl) was much lower, and it was only 2.3% (Table 8, No. 5), which might be due to the passivation of the benzene ring with the electron-withdrawing group. The conversion rate reached 21.6% (Table 8, No. 6), which might be due to the formation of a π–π conjugate bond in the aromatic ketone and was conducive to the reaction.

### 3.9. Effect of the Light Wavelength on the Reaction

This experiment investigated the effect of six different light wavelengths on the reaction. The reaction conditions were the same as those above.

**Figure 8:**
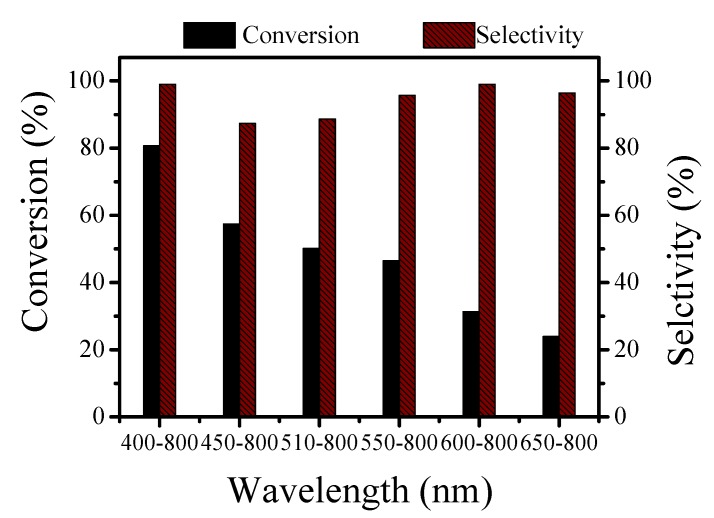
Effect of different light wavelengths on the reaction of benzyl alcohol and acetophenone.

The visible wavelength range was wider, and the conversion of the alkylation reaction was higher. When the wavelength range was 400–800 nm, the conversion rate was up to 80.7%, and the selectivity was close to 100%. When the wavelength range gradually became narrower, the conversion rate decreased, which was consistent with the results of the UV-vis DRS analysis (Figure 8).

### 3.10. Effect of the Light Intensity on the Reaction

The effect of light intensity on the alkylation reaction was investigated under optimal conditions. The reaction conditions were the same as those above. A 4 wt % Pt/γ-Bi_2_MoO_6_ sample was used to explore the effects of six different light intensities on the reaction.

**Figure 9:**
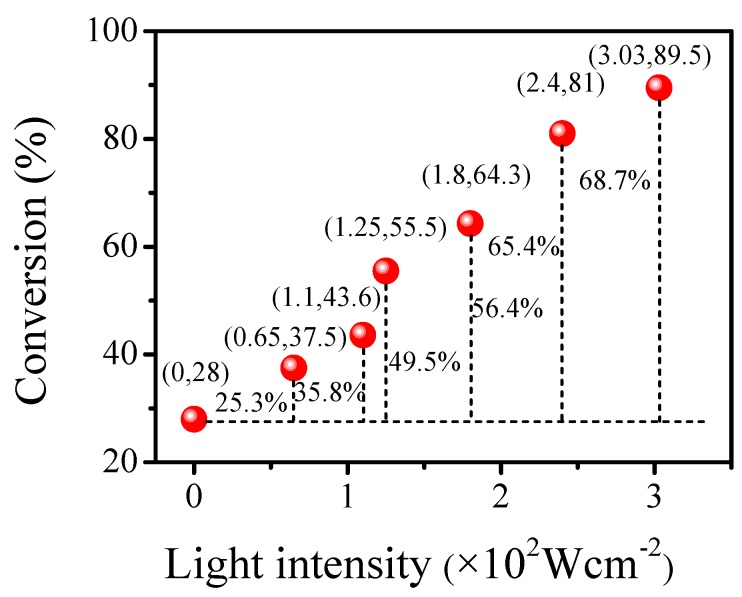
Effect of different light intensities on the reaction of benzyl alcohol and acetophenone.

With increasing light intensity, the conversion rate of the alkylation reaction gradually increased. When the light intensity was 0, the conversion rate was 28%, which was a thermal reaction. When the light intensity was 0.00303 W·cm^−2^, the conversion rate was as high as 89.5%. Excluding the conversion rate of the thermal reaction, the contribution rate of the light reaction reached 61.5%. Visible light is a necessary condition for the reaction to proceed (Figure 9).

### 3.11. Cycling Capability Test of the Catalyst

Under the optimal conditions, 4 wt % Pt/γ-Bi_2_MoO_6_ was used to test the cycling ability, and five cycling experiments were performed to investigate the stability of the photocatalyst.

**Figure 10:**
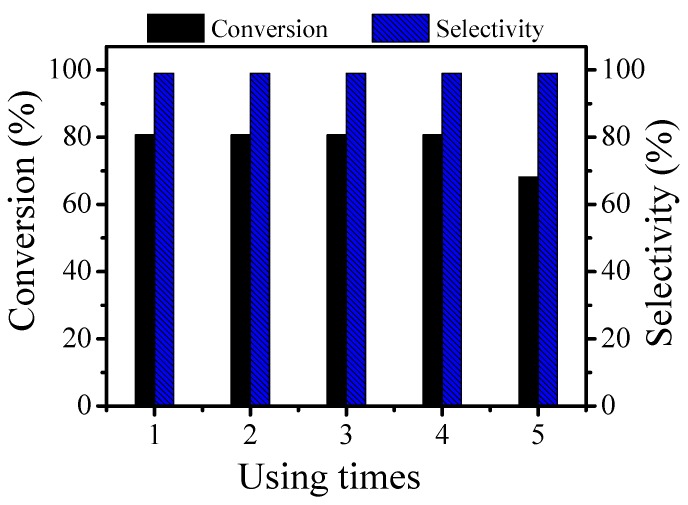
The recycling ability test of 4 wt % Pt/γ-Bi_2_MoO_6_ on the reaction of benzyl alcohol and acetophenone.

During the five cycles, the photocatalyst was stable. The photocatalyst could still maintain good activity during the first four cycles. The conversion rate of the photocatalyst significantly decreased during the fifth cycle, although, the selectivity remained stable. From the SEM and TEM images of the recycled 4 wt % Pt/γ-Bi_2_MoO_6_ catalyst, it can be seen that agglomeration occurred after five cycles with the catalyst. Thus, the number of active sites on the surface of the catalyst decreased, and therefore, the conversion rate decreased (Figure 10).

### 3.12. Effect of Reactive Groups on the Mechanism

**Figure 11:**
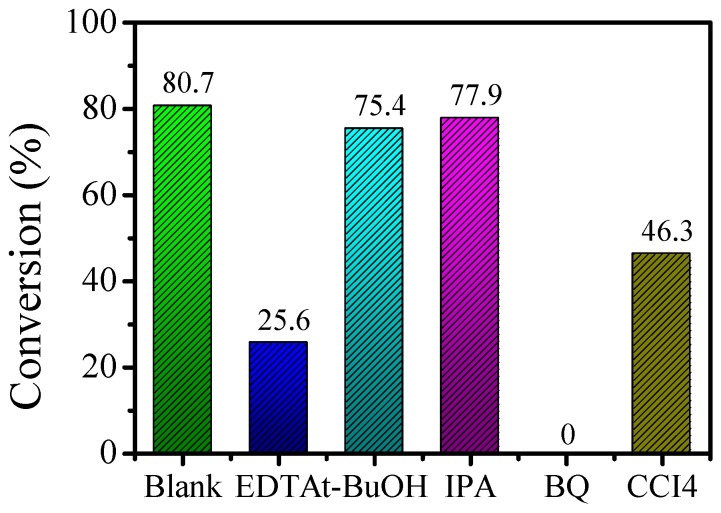
Effects of scavengers on the reaction of benzyl alcohol and acetophenone.

To explore the active species of the alkylation reaction, the above optimal conditions were selected. Ethylenediamine tetraacetic acid (EDTA), t-BuOH, isopropanol alcohol (IPA), 1,4-p-benzoquinone (BQ), and carbon tetrachloride (CCl_4_) were used as the h^+^, •OH, •O_2_^−^, and e^−^ quenchers, respectively. The results are shown in Figure 11.

Using a blank experiment as a reference, the conversion rate of 1,4-p-benzoquinone (BQ) was 0. The above result indicates that the reaction could not proceed after adding the 1,4-p-benzoquinone (BQ) quencher, thus proving that superoxide radicals (•O_2_^−^) are active species. The conversion rate of the reaction was basically unchanged after the addition of t-BuOH and isopropanol alcohol (IPA), indicating that hydroxyl radicals (•OH) are not active species in the reaction. The conversion rates after adding ethylenediamine tetraacetic acid (EDTA) and carbon tetrachloride (CCl_4_) were 25.6% and 46.3%, respectively. This indicates that during the α-alkylation reaction, holes (h^+^) and electrons (e^−^) act as active species. 

## 4. Conclusions

Bi(NO_3_)_3_·5H_2_O and (NH_4_)_6_Mo_7_O_2_·4H_2_O were used as raw materials, and pure flaky bismuth molybdate was successfully prepared in different thicknesses by a simple hydrothermal synthesis method. Pt was successfully loaded on the support by a NaBH_4_ reduction method. According to SEM and TEM, the thinner bismuth molybdate nanosheets are, the better the performance of the photocatalyst is. The UV-vis DRS results show that the Pt/γ-Bi_2_MoO_6_ photocatalyst had a strong absorption peak within the visible light range. The catalyst had good optical properties, which could effectively reduce the possibility of electron-hole recombination, and could modify the deficiency of Pt/γ-Bi_2_MoO_6_ by PL.

Pt could act as an electron trapping agent, and the use of 4 wt % Pt/γ-Bi_2_MoO_6_ prepared by the nitric acid method (pH = 9, reaction temperature 180 °C) was shown to be the best catalyst when applied to the alkylation reaction of benzyl alcohol and acetophenone. The reaction was investigated by studying a series of variables. The photocatalyst was stable after five cycles.

## Figures and Tables

**Table 1:** Specific surface areas of the different catalysts.

Variate	Sample BET	Surface Area (m^2^·g^−1^)
	Nitric acid	3.26
Preparation method	Ethylene glycol	2.71
	Acetic acid	13.08
	pH = 8	3.15
pH value	pH = 9	3.26
(Nitric acid method)	pH = 10	4.39
	pH = 11	6.28
Hydrothermal temperature (Nitric acid method)	130 °C	2.65
	150 °C	3.08
	180 °C	3.26
Different mass fraction	1 wt % Pt	5.27
	2 wt % Pt	7.85
	3 wt % Pt	13.45
	4 wt % Pt	19.63
	5 wt % Pt	17.35

**Table 2:** Effect of the different catalysts on the reaction of benzyl alcohol and acetophenone.

No.	Catalyst	Under Visible Light		In the Dark	
		Conv. (%)	Sel. (%)	Conv. (%)	Sel. (%)
1	BMO (N, pH = 9, 180 °C)	37.6	>99	1.1	>99
2	BMO (E, pH = 9, 180 °C)	15.2	>99	0.5	>99
3	BMO (A, pH = 9, 180 °C)	5.7	>99	0.1	>99
4	BMO (pH = 8, N, 180 °C)	24.3	>99	0.5	>99
5	BMO (pH = 10, N, 180 °C)	35.5	>99	0.8	>99
6	BMO (pH = 11, N, 180 °C)	29.4	>99	0.6	>99
7	BMO (150 °C, N, pH = 9)	32.1	>99	0.9	>99
8	BMO (130 °C, N, pH = 9)	27.2	>99	0.8	>99
9	1 wt % Pt/BMO (N)	44.8	>99	1.7	>99
10	2 wt % Pt/BMO (N)	49.4	>99	2.2	>99
11	3 wt % Pt/BMO (N)	50.7	>99	3.5	>99
12	4 wt % Pt/BMO (N)	55.9	>99	5.2	>99
13	5 wt % Pt/BMO (N)	43.3	>99	1.9	>99

Note: BMO stands for γ-Bi_2_MoO_6_. N stands for γ-Bi_2_MoO_6_ (nitric acid method, pH = 9, 180 °C), E stands for γ-Bi_2_MoO_6_ (ethylene glycol method, pH = 9, 180 °C), and A stands for γ-Bi_2_MoO_6_ (glacial acetic method, pH = 9, 180 °C).

**Table 3:** Effect of different amounts of catalyst on the reaction of benzyl alcohol and acetophenone.

No.	Catalyst Amount (mg)	Under Visible Light		In the Dark	
		Conv. (%)	Sel. (%)	Conv. (%)	Sel. (%)
1	0	0.9	>99	0	0
2	25	27.2	>99	1.4	>99
3	50	55.9	>99	5.2	>99
4	75	65.3	>99	7.8	>99
5	100	57.5	>99	5.4	>99

**Table 4:** Effect of different solvents on the reaction of benzyl alcohol and acetophenone.

No.	Solvent	Polar Index	Under Visible Light		In the Dark	
			Conv. (%)	Sel. (%)	Conv. (%)	Sel. (%)
1	DMSO	7.2	0.5	>99	0	0
3	DMF	6.4	7.2	>99	0	0
4	Acetonitrile	6.2	11.2	>99	0.3	>99
5	1,4-Dioxane	4.8	45.3	>99	1.7	>99
6	Isopropanol	4.3	49.1	>99	4.5	>99
7	Tetrahydrofura	4.2	20.1	>99	0.9	71.8
8	Mesitylene	1.9	5.7	>99	0	0
9	Cyclohexane	0.1	7.3	>99	0	0
10	n-Heptane	0.06	65.3	>99	7.8	>99
11	Petroleum ether	0.01	17.5	>99	0.4	>99
12	n-Hexane	0	36.2	>99	3.8	>99

**Table 5:** Effect of the different bases on the reaction of benzyl alcohol and acetophenone.

No.	Base	Under Visible Light		In the Dark	
		Conv. (%)	Sel. (%)	Conv. (%)	Sel. (%)
1	NaOH	65.3	>99	7.8	>99
2	KOH	27.2	94.5	0.5	36.2
3	LiOH	30.7	91.2	4.1	67.3
4	Na_2_CO_3_	5.7	87.3	0	0
5	K_2_CO3	3.8	97.9	0.4	21.7
6	CH_3_ONa	25.1	>99	0.6	>99
7	Cs_2_CO_3_	19.3	>99	0.3	40.1
8	Blank	0.5	94.3	0	0

**Table 6:** Effect of different amounts of base on the reaction of benzyl alcohol and acetophenone.

No.	Base Amount (mmol)	Under Visible Light		In the Dark	
		Conv. (%)	Sel. (%)	Conv. (%)	Sel. (%)
1	0	1.3	>99	0	0
2	0.6	14.3	>99	1.5	>99
3	0.8	26.7	>99	1.4	>99
4	1.0	65.3	>99	7.8	>99
5	1.2	71.7	>99	8.2	>99
6	1.4	57.5	>99	5.4	>99

**Table 7:** Effect of different amounts of reactants on the reaction of benzyl alcohol and acetophenone.

No.	Benzyl Alcohol (mmol)	Benzyl Acetophenone (mmol)	Under Visible Light		In the Dark	
			Conv. (%)	Sel. (%)	Conv. (%)	Sel. (%)
1	1.0	1.0	71.7	>99	8.2	>99
2	2.0	1.0	76.5	>99	8.7	>99
3	3.0	1.0	80.7	>99	9.3	>99
4	4.0	1.0	74.7	>99	7.9	>99
5	3.0	2.0	36.5	>99	4.9	>99
6	3.0	3.0	21.9	>99	3.2	>99

**Table 8:** Effect of the different alcohol and acetophenone derivatives on the reaction of benzyl alcohol and acetophenone.

No.	Alcohol	Acetophenone	Under Visible Light		In the Dark	
			Conv. (%)	Sel. (%)	Conv. (%)	Sel. (%)
1	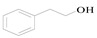	Benzyl acetophenone	10.2	88.5	0	0
2	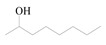	Benzyl acetophenone	33.7	>99	0	0
3	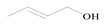	Benzyl acetophenone	1.7	88.5	0	0
4	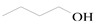	Benzyl acetophenone	1.9	>99	0	0
5	Benzyl alcohol	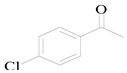	2.3	>99	0	0
6	Benzyl alcohol	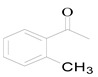	21.6	>99	0	0
7	Benzyl alcohol	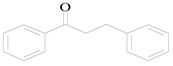	15.4	>99	0	0
8	Benzyl alcohol	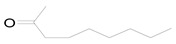	9.1	>99	0	0

## Supplementary Materials

The following are available online at https://www.mdpi.com/2079-4991/10/4/646/s1.

## References

[1] Bai M.F., Xin H., Guo Z., Guo D.P., Wang Y., Zhao P., Li J.Y. (2017). α-Alkylation of ketones with primary alcohols driven by visible light and bimetallic gold and palladium nanoparticles supported on transition metal oxide. Appl. Surf. Sci..

[2] Liu P.C., Liang R., Lu L., Yu Z.T., Li F. (2017). Use of a cyclometalated iridium(III) complex containing a NCN-coordinating terdentate ligand as a catalyst for the α-alkylation of ketones and N-alkylation of amines with alcohols. J. Org. Chem..

[3] Xing Y.X., Gao X.C., Ji G.F., Liu Z.L., Du C.F. (2019). Synthesis of carbon doped Bi_2_MoO_6_ for enhanced photocatalytic performance and tumor photodynamic therapy efficiency. Appl. Surf. Sci..

[4] Xu J.J., Yue J.P., Niu J.F., Chen M.D. (2019). Synergistic removal of Cr(VI) and dye contaminants by 0D/2D bismuth molybdate homojunction photocatalyst under visible light. Appl. Surf. Sci..

[5] Chen D.M., Hao Q., Wang Z.H., Hao D., Zhu Y.F. (2016). Influence of phase structure and morphology on the photocatalytic activity of bismuth molybdates. Cryst. Eng. Commun..

[6] Shi C.J., Dong X.L., Hao Y.C., Wang X.Y., Ma H.C., Zhang X.F. (2017). The controllable fabrication of a novel hierarchical nanosheet-assembled Bi_2_MoO_6_ hollow micronbox with ultra-high surface area for excellent solar to chemical energy conversion. RSC Adv..

[7] Guo C.S., Xu J., Wang S.F., Zhang Y., He Y., Li X.C. (2013). Photodegradation of sulfamethazine in an aqueous solution by a bismuth molybdate photocatalyst. Catal. Sci. Technol..

[8] Song L.N., Chen L., He J.P., Chen P., Zeng H.K., Au C.T., Yin S.F. (2017). The first synthesis of Bi self-doped Bi_2_MoO_6_–Bi_2_Mo_3_O_12_ composites and their excellent photocatalytic performance for selective oxidation of aromatic alkanes under visible light irradiation. Chem. Commun..

[9] Dai Z., Qin F., Zhao H.P., Tian F., Liu Y.L., Chen R. (2016). Time-dependent evolution of the Bi_3.64_Mo_0.36_O_6.55_/Bi_2_MoO_6_ heterostructure for enhanced photocatalytic activity viathe interfacial hole migration. Cryst. Eng. Commun..

[10] Zhang J.L., Zhang L.S., Yu N., Xu K.B., Li S.J., Wang H.L., Liu J.S. (2015). Flower-like Bi_2_S_3_/Bi_2_MoO_6_ heterojunction superstructures with enhanced visible-light-driven photocatalytic activity. RSC Adv..

[11] Wang X., Gu L.L., Fang G.L., Wang X. (2013). A facile mixed-solvothermal route to Bi_2_MoO_6_ nanoflakes and their visible-light-responsive photocatalytic activity. Mater. Res. Bull..

[12] Li Z.F., Wu Z.H., Zhang S.M., Shen J., Feng W.H., Du Y., Wan L., Zhang S.H. (2018). Defect state of indium-doped bismuth molybdate nanosheets for enhanced photoreduction of chromium(VI) under visible light illumination. Dalton Trans..

[13] Guo C.S., Xu J., Wang S.F., Li L., Zhang Y., Li X.C. (2012). Facile synthesis and photocatalytic application of hierarchical mesoporous Bi_2_MoO_6_ nanosheet-based microspheres. Cryst. Eng. Commun..

[14] Chanapa K.M., Vladimir M., Annick R., Caroline P. (2009). Elucidating the genesis of Bi_2_MoO_6_ catalyst by combination of synchrotron radiation experiments and Raman scattering. Chem. Commun..

[15] Li H.D., Li W.J., Gu S.N., Wang F.Z., Zhou H.L. (2016). In-built Tb^4+^/Tb^3+^ redox centers in terbium-doped bismuth molybdate nanograss for enhanced photocatalytic activity. Catal. Sci. Technol..

[16] Zhang Z.W., Jin S.S., Hao H.S., Hou Y.X., Hou H.M., Zhang G.L., Bi J.R., Yan S., Liu G.S., Gao W.Y. (2019). Synthesis and enhanced visible-light photocatalytic activity of Tm^3+^/Yb^3+^ co-doped Bi_3.64_Mo_0.36_O_6.55_. Chem. Select..

[17] Jung J.C., Kim H.S., Choi Y.M., Chung T.J., Kim S.J., Lee S., Song I.K. (2007). Preparation and characterization of bismuth molybdate catalyst for oxidative dehydrogenation of n-butene into1,3-butadiene. Mater. Sci..

[18] Wen J., Zhang S.B., Shi N.F., Liao X.M., Yin G.F., Huang Z.B., Chen X.C., Pu X.M. (2019). Facile synthesis of a Bi_2_MoO_6_/TiO_2_ nanotube arrays composite by the solvothermal method and its application for high-performance supercapacitor. RSC Adv..

[19] Li H.Z., Sun B., Xu Y.C., Qiao P.Z., Wu J.X., Pan K.G., Tian H., Wang L., Hou W.Z. (2018). Surface defect-mediated efficient electron-hole separation in hierarchical flower-like bismuth molybdate hollow spheres for enhanced visible-light-driven photocatalytic performance. J. Colloid. Interface Sci..

[20] Liang D.Y., Ding Y., Wang N., Cai X.M., Li J., Han L.Y., Wang S.Q., Han Y.Y., Jia G., Wang L.Y. (2017). Solid-state reaction synthesis for mixed-phase Eu^3+^-doped bismuth molybdate and its luminescence properties. Mod. Phys. Lett. B.

[21] Li H.Z., Sun B.J., Xu Y.C., Qiao P.Z., Wu J.X., Pan K., Tian G.H., Wang L., Hou W.Z. (2018). Construction of a Bi_2_MoO_6_:Bi_2_Mo_3_O_12_ heterojunction for efficient photocatalytic oxygen evolution. Chem. Eng. J..

[22] Wu X.L., Hart N., Wen X.M., Wang L., Du Y., Dou S.X., Rose A., Jason S. (2018). Improving the photo-oxidative performance of Bi_2_MoO_6_ by harnessing the synergy between spatial charge separation and rational co-catalyst deposition. ACS Appl. Mater..

[23] Zhang B., Yang X.J., Li J., Cheng G. (2018). Selective aerobic oxidation of alkyl aromatics on Bi_2_MoO_6_ nanoplates decorated with Pt nanoparticles under visible light irradiation. Chem. Commun..

[24] Meng X.C., Zhang Z.S. (2017). Pd-doped Bi_2_MoO_6_ plasmonic photocatalysts with enhanced visible light photocatalytic performance. Appl. Surf. Sci..

[25] Wang M., Zhang Y., Jin C.Y., Li Z.L., Chai T.Y., Zhu T. (2019). Fabrication of novel ternary heterojunctions of Pd/g-C_3_N_4_/Bi_2_MoO_6_ hollow microspheres for enhanced visible-light photocatalytic performance toward organic pollutant degradation. Sep. Purif. Technol..

[26] Bi J.H., Fang L., Li X.F., Liu M., Liang S.J., Zhang Z.Z., He Y.H., Lin H.X., Wu L., Liu S.W. (2015). Ternary reduced-graphene-oxide/Bi_2_MoO_6_/Au nanocomposites with enhanced photocatalytic activity under visible light. J. Alloys Compd..

[27] Ma Y., Jia Y.L., Lin Y.H., Shi W.B. (2019). Hierarchical Ag/Bi_2_MoO_6_ hollow nanoboxes with high photocatalytic performance. Dalton. Trans..

[28] Jia Z., Yu F.L., Zhang L.C., Zeng S., Liang S.X., Li Y.Y., Lu J. (2019). Pt nanoparticles decorated heterostructured g-C_3_N_4_/Bi_2_MoO_6_ microplates with highly enhanced photocatalytic activities under visible light. Sci. Reports..

[29] Li X., Su M.Y., Zhu G.F., Zhang K.G., Zhang X., Fan J. (2018). Fabrication of a novel few-layer WS_2_/Bi_2_MoO_6_ plate-on-plate heterojunction structure with enhanced visible-light photocatalytic activity. Dalton Trans..

[30] Wu J., Sun Y.Y., Gu C.H., Wang T., Xin Y.J., Chai C., Cui C.Y., Ma D. (2018). Pt supported and carbon coated Bi_2_MoO_6_ composite for enhanced 2,4–dibromophenol degradation under visible-light irradiation: Insight into band gap structure and photocatalytic mechanism. Appl. Catal B Environ..

[31] Deng M.S., Huan Y. (2019). Cryogenic ball milling synthesis of Bi_2_MoO_6_/FePt and Bi_2_MoO_6_/Pt composites and the comparison of their photocatalytic characteristics. Ceram. Int..

[32] Li H.H., Li K.W., Hao W. (2009). Hydrothermal synthesis and photocatalytic properties of bismuth molybdate materials. Mater. Chem. Phys..

