# Supplementary material for: Preparation of Pt/γ-Bi2MoO6 Photocatalysts and Their Performance in α-Alkylation Reaction under Visible Light Irradiation
Source: Nanomaterials (Basel). 2020 Mar 30;10(4):646. doi: 10.3390/nano10040646 (PMC7221669; doi:10.3390/nano10040646)

Supporting Information

Preparation of Pt/γ-Bi_2MoO_6 Photocatalysts and Their Performance in α-Alkylation Reaction under Visible Light Irradiation

Haiying Li [1], Xiujuan Yu [1,2], Xueli Hao [1], Zhiying Zhang [1], Yan Wang [1] and Jingyi Li [1,*]

[1] College of Chemistry and Chemical Engineering, Inner Mongolia University, Hohhot 010021, China; lihaiying0528gsj@163.com (H.L.); yuxiujuan810@163.com (X.Y.); hxl18747721020@163.com (X.H.); zhangzhiying2019@163.com (Z.Z.); wynmgnhy@163.com (Y.W.)

[2] Hebei Key Laboratory of Neuropharmacology, Hebei North University, Zhangjiakou 075000, China

* Correspondence: 111972266@imu.edu.cn; Tel.: +86-138-4812-9221

Received: 3 March 2020; Accepted: 29 March 2020; Published: date

Analysis of GC-MS:

A mixture of n-heptane (6 mL), benzyl alcohol (3 mmol), acetophenone (1 mmol), a base of NaOH (1.2 mmol), and a catalyst of Bi_2MoO_6 (nitric acid method, pH = 9, 180 °C) (75 mg), under the condition of 400–800 nm filter, air atmosphere for 24 h, a light source intensity of 2.0 × 10^{-2}W·cm^{-2}, a reaction temperature of 30 ± 3 °C. The mixture was detected by GC-MS.

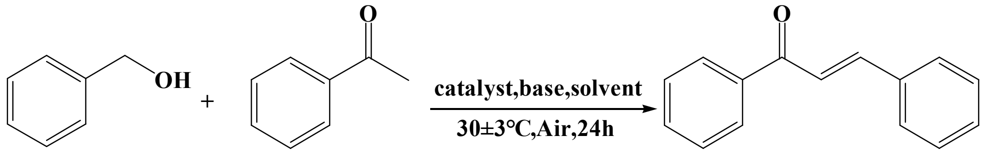

Results of GC-MS:

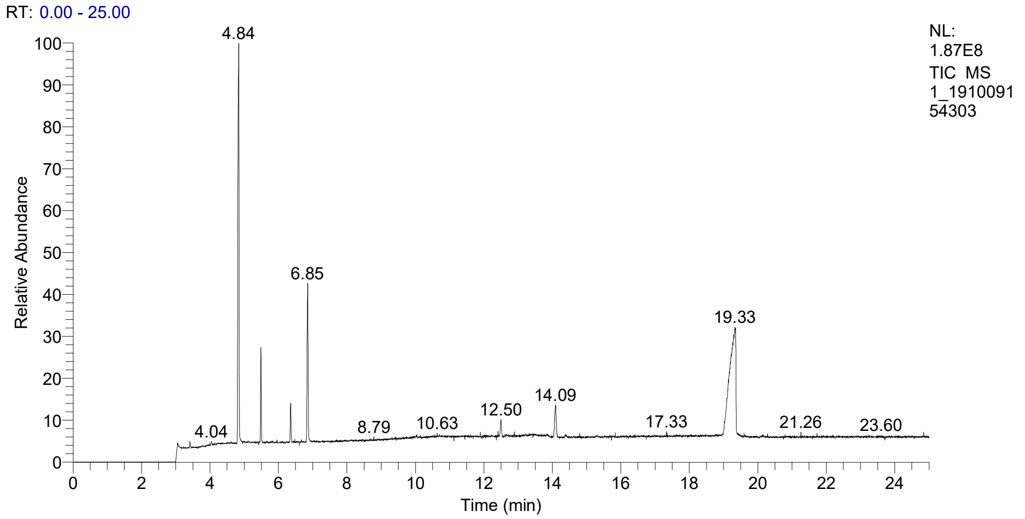

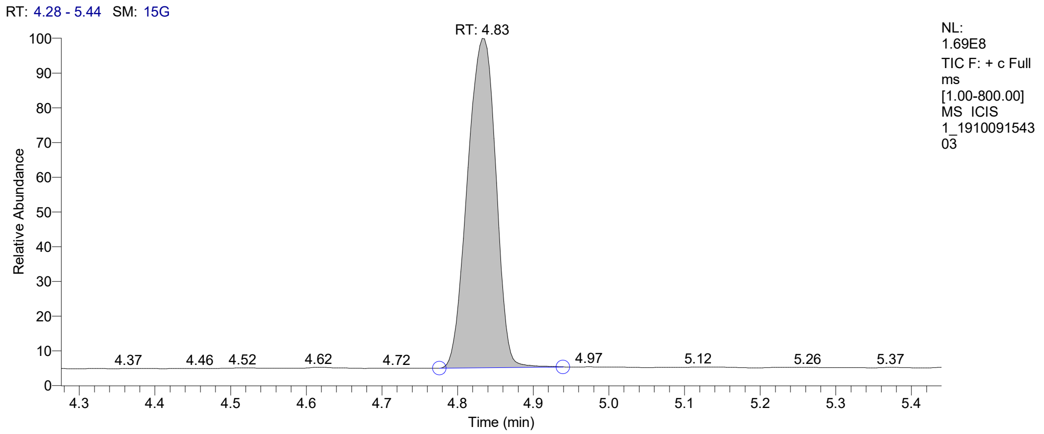

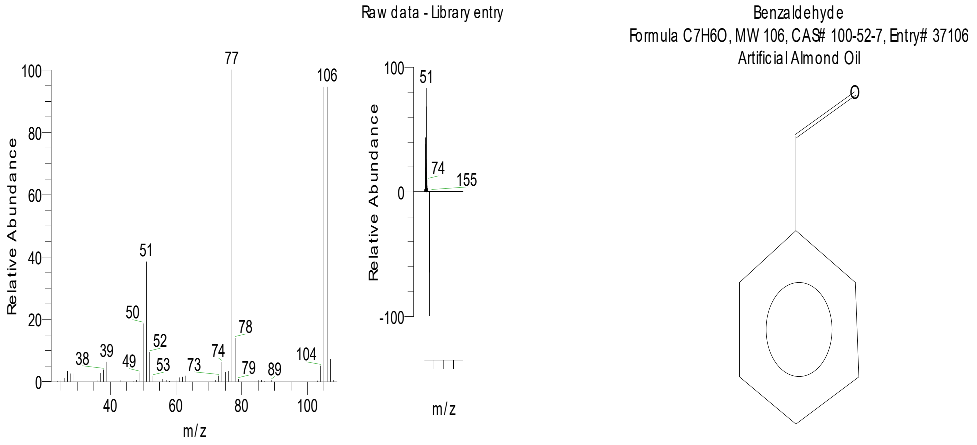

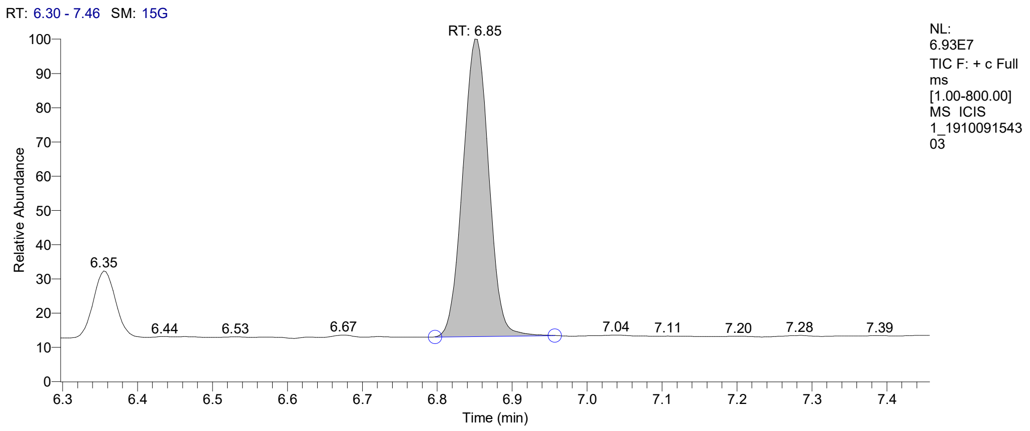
RT: 6.30 - 7.46 SM: 15G
RT: 6.85
NL:
6.93E7
TIC F: + c Full
ms
[1.00-800.00]
MS ICIS
1_1910091543
03
6.35
6.44
6.53
6.67
7.04
7.11
7.20
7.28
7.39
Relative Abundance
Time (min)

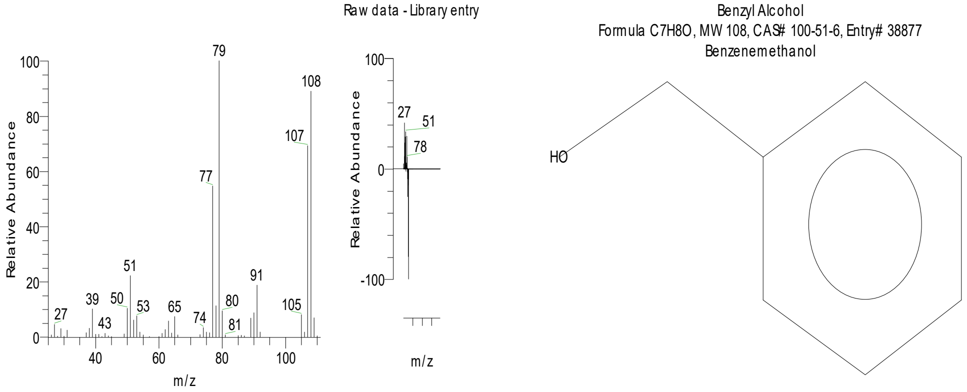
Raw data - Library entry
Benzyl Alcohol
Formula C7H8O, MW 108, CAS# 100-51-6, Entry# 38877
Benzenemethanol
HO
Relative Abundance
m/z
27
39
43
50
51
53
65
74
77
79
80
81
91
105
107
108
27
51
78

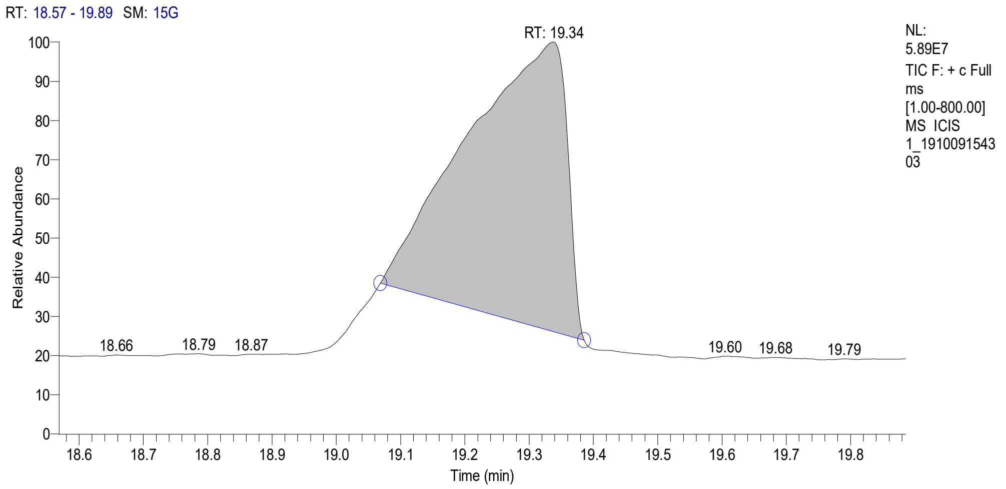
RT: 18.57 - 19.89 SM: 15G
RT: 19.34
NL:
5.89E7
TIC F: + c Full
ms
[1.00-800.00]
MS ICIS
1_1910091543
03
18.66
18.79
18.87
19.60
19.68
19.79
Relative Abundance
Time (min)

Raw data - Library entry

2-Propen-1-one, 1,3-diphenyl-
Formula C15H12O, MW 208, CAS# 94-41-7, Entry# 37600
Chalcone

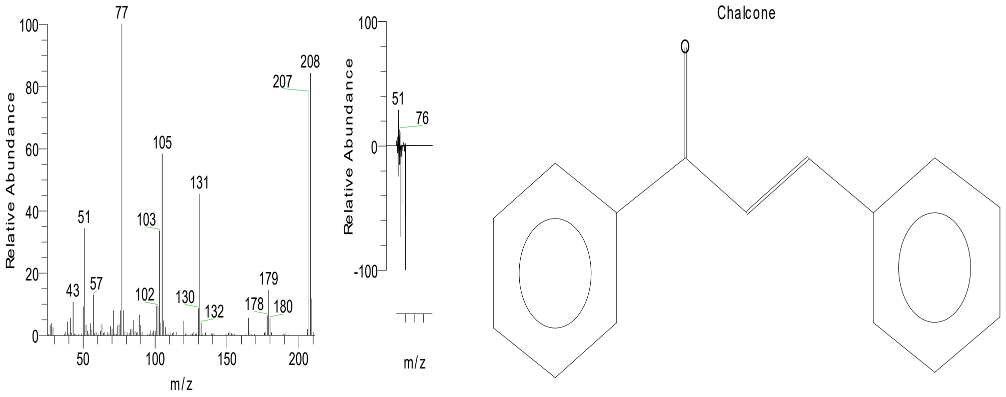

Supplement: Supplementary file 1 [file nanomaterials-10-00646-s001.pdf]
